# Teacher Autonomy Support and Internalizing Problems of Adolescents from Divorced and Intact Families: Moderation by Personality Typologies

**DOI:** 10.1007/s10578-022-01392-x

**Published:** 2022-07-05

**Authors:** Xiaoyu Lan, Stefanos Mastrotheodoros

**Affiliations:** 1 Promenta Research Center, Department of Psychology, University of Oslo, Oslo, Norway; 2 Department of Psychology, University of Crete, Rethymno, Greece; 3 Department of Youth and Family, Utrecht University, Utrecht, the Netherlands

**Keywords:** Internalizing problems, Teacher autonomy support, Personality profiles, Parental divorce, Adolescents

## Abstract

The present research compared internalizing problems of adolescents who experienced parental divorce with those of adolescents who remained in intact families. Furthermore, this research investigated the association of teacher autonomy support with adolescents’ internalizing problems for the whole sample and further ascertained whether this association was moderated by distinctive personality profiles using a person-centered approach and family structures (divorced vs. intact families). A sample of 2756 Chinese adolescents (8.5% from divorced families), aged 13–18 years, participated in the present research. They completed a set of self-reported questionnaires during school hours. Results based on ANCOVA showed that adolescents who experienced parental divorce reported higher internalizing problems than did those who remained in intact families. Moreover, latent profile analysis revealed three personality profiles: psychopathic (22.7%), normative (56.4%), and resilient (20.9%). In addition, teacher autonomy support was negatively related to adolescents’ internalizing problems in the overall sample. However, interaction analyses further exhibited that this association was insignificant for psychopathic adolescents who experienced parental divorce. The current findings indicate that although teacher autonomy support may protect adolescents from internalizing problems, psychopathic adolescents whose parents got divorced should be paid exceptional attention by mental health professionals and school counselors.

## Introduction

Internalizing problems refer to disturbances in emotion, such as depressive and anxiety symptoms [1]. Adolescents have been demonstrated to be highly prone to exhibiting internalizing problems, which have long-term implications for their maladaptive emotional-behavioral functions in adolescence and beyond [2–4]. Thus, an investigation focusing on the correlates of internalizing problems during adolescence would be essential to inform targeted intervention or prevention programs.

Past research has demonstrated that parental divorce is associated with an increased risk for emotional and behavioral difficulties, including internalizing problems [5–9]. In contrast to this dominant view, mounting evidence has shown no significant differences in adolescents’ post-divorce adjustment, as compared with those adolescents who remained in intact families [10, 11]. Indeed, adolescents’ responses to parental divorce show salient variability. Given the increasing divorce rates globally and the contrasting findings on post-divorce adjustment of adolescents, we aimed to confirm the differences in internalizing problems between adolescents from divorced and intact families that existing research has shown.

Furthermore, we used a socioecological framework to investigate the correlates of adolescents’ internalizing problems [12]. Specifically, this framework postulates that human development is embedded in an interactive system in which environmental and personal factors interact . Specifically, this study focused on environmental variables, such as family structures and teacher autonomy support, and personal variables, such as personality traits. According to this framework, these variables interact with each other to explain individual differences in internalizing problems. 

## Teacher autonomy support

Youth spend most of their time in school during adolescence, and teachers play a critical role in their various domains [13, 14]. In the Chinese school system, one head teacher is usually in charge of the same classroom, including students’ educational, administrative, and daily affairs, and thus becomes very familiar with those students [15]. Given the salient role of the head teacher in adolescents’ social sphere, the current study focused on perceived autonomy support, particularly from the head teacher in each classroom.

Teacher autonomy support refers to a type of teaching style in which teachers use non-controlling language, provide a meaningful rationale and choice, acknowledge students’ negative feelings, and nurture students’ motivational resources [16, 17]. Based on the self-determination theory [18], individuals are inherently proactive, and a social context that facilitates autonomy and self-motivation could forge a better sense of well-being and personal growth. Following this theoretical perspective, empirical studies have shown that teacher autonomy support is inversely related to negative affect in adolescents [19]. This is mainly because the autonomy-supportive motivating style in the classroom promotes engagement and encourages adolescents to explore independently. This way, a sense of control and mastery is engendered, which enhances adolescents’ adaptive functioning. Yu et al. (2016) also reported a negative relationship between teacher autonomy support and Chinese adolescents’ anxiety and depressive symptoms [14]. According to the self determination theory and empirical studies reviewed above, we hypothesized that teacher autonomy support would be negatively related to adolescents’ internalizing problems.

Furthermore, although supervised by the same teachers, not every student in the same classroom would exhibit similar levels of internalizing difficulties. It is generally assumed that internalizing problems are not only driven by environmental influences, such as teacher autonomy support, but are also impacted by personal traits [12, 20]. We, therefore, investigated the role of personality traits in the proposed study association, as elaborated upon below.

## Personality traits

Among existing theoretical frameworks concerning personality traits, the Big Five Model has probably received the most robust empirical support [21]. The Big Five framework points out that most individual differences in personality can be classified into five empirically derived dimensions: openness, conscientiousness, extraversion, agreeableness, and neuroticism. Previous research has predominantly applied a variable-centered approach to study the association of these personality traits with other variables, based on the assumptions that populations are homogeneous [22, 23]. Despite this, because personality comprises not the sum of segregated personality characteristics but rather constellations of traits, a person-centered approach might offer a more real-life view of how personality plays in people’s lives.

To this end, we aimed at applying Block and Block’s (1980) framework, which postulates that three types of personality parsimoniously explain relatively stable individual differences [24]. These personality types are the resilient, the undercontrolling, and the overcontrolling people. Resilient people are described as being balanced and possessing high adaptive resources, and they score low in neuroticism and high in the remaining dimensions; undercontrollers are described as having little impulse control, and they score low in agreeableness and conscientiousness; overcontrollers are characterized by acting primly and being emotionally brittle and inexpressive, and they score high in neuroticism and low in extraversion [25, 26]. Xie and his colleagues (2016) replicated these personality types in Chinese adolescents and found that resilient adolescents have good social adaptability, whereas undercontrollers exhibit high levels of aggression. Despite employing such an approach to derive personality profiles, previous studies have not reached a consensus concerning the number and characteristics of personality profiles [23]. We, therefore, aimed at further confirming whether it is possible to empirically derive three personality profiles based on a large sample of Chinese adolescents.

Furthermore, while previous studies employed a person-centered approach to derive personality typologies and then investigated their associations with variables of interest, the moderating role of such personality profiles in the association of teacher autonomy support with adolescents’ internalizing problems has not been examined so far. Employing such an approach would allow us to derive unobserved subgroups of adolescents characterized by different natural configurations of personality characteristics and explore whether specific subgroups of adolescents are less responsive to teacher autonomy support and thus become more vulnerable to internalizing problems than others. That said, hinging upon distinctive personality profiles, adolescents may perceive autonomy support provided by their teachers differently, which in turn might influence the association of teacher autonomy support with adolescents’ internalizing problems. For example, overwhelming evidence has shown that socially undesirable personality traits (e.g., high neuroticism) are strongly related to the heightened negative perception of environmental influences and thus becoming overresponsive to stress, whereas socially desirable personality traits (e.g., high extraversion) are related to more positive perceptions and adaptive emotional patterns when confronting stressful experiences [27, 28].

## The present research

The present study aimed to answer three research questions (RQ). RQ1: Are there any differences in internalizing problems between adolescents from divorced families and their peers who remained in intact families? RQ2: Is it possible to identify personality profiles using a person-centered approach? RQ3: What is the association of teacher autonomy support with adolescents’ internalizing problems on a combined sample of adolescents from divorced and intact families? Could emerging personality profiles and family structures explain individual differences in this association?

Based on the current literature review, three hypotheses with respect to each RQ were generated.

### Hypothesis 1

Adolescents whose parents got divorced would report higher internalizing problems than their peers who remained in intact families.

### Hypothesis 2

Three personality profiles might be revealed: resilients, undercontrollers, and overcontrollers.

### Hypothesis 3

Teacher autonomy support would be negatively related to adolescents’ internalizing problems for the whole sample. In terms of the potential moderating role of personality profiles and family structures, we did not generate a priori hypothesis due to the scarcity of literature in this field and the complexity of two- and three-way interactive patterns. Nevertheless, based on the current literature review, some exploratory expectations might be made. For instance, we expected that this association might be more potent for adolescents from divorced families than for those from intact families due to diminished parental involvement after divorce, and/or this association might be pronounced in adolescents with socially desirable personality profiles (e.g., resilient) than others, as these adolescents are more likely to perceive positive environmental influence and thus report less internalizing difficulties; and finally, perhaps this association might be heightened in post-divorce adolescents with more socially desirable personality profiles than in their corresponding counterparts. That said, post-divorce adolescents with socially desirable personality profiles might particularly benefit from high teacher autonomy support, as they often have restricted social and family resources.

## Method

### Participants and procedures

Prior to data collection, ethical approval was granted from the ethics review board at the Northwest Minzu University. Through school collaborations, eight secondary schools agreed to participate in the present study. After receiving the confirmation from school principals, head teachers in each classroom helped us send an information flyer and an informed consent form to students’ parents/guardians online. At the same time, adolescents were asked whether they were willing to participate in this investigation, in which confidentiality, anonymity, and the right to withdraw at any time were guaranteed. To ensure reliability, instruments that had previously been validated in Chinese adolescents were carefully selected. During school hours, graduate students, together with head teachers, supervised adolescents’ completion of a few self-reported questionnaires written in simplified Chinese during a regular class hour (approximately 45 min).

A sample of 2756 adolescents participated in the current study. Of these participants, 235 adolescents (66.3% girls; *M*_age_ = 15.56; *SD* = 1.58) were from divorced families, and 2521 adolescents (55.1% girls; *M*_age_ = 15.66; *SD* = 1.57) were from intact families. For adolescents from divorced families, 23.8%, 29.4%, and 9.4% of adolescents resided with their fathers, mothers, and grandparents, respectively; for the remaining adolescents (37.4%), their parents shared custody. The average child age when parents divorced was 7.71 years (*SD* = 3.98), and parents got divorced on average 7.91 years (*SD* = 4.05) before the study took place. Preliminary analyses showed that these two groups did not significantly differ in age (*t* = 0.95, *df* = 2754, *p* = .34) or family socioeconomic status (*t* = 0.16, *df* = 2754, *p* = .86), but adolescents who experienced parental divorce were more often girls than adolescents who remained in intact families (*χ*^2^ = 11.03, *df* = 1, *p* < .001).

## Measures

### Internalizing problems

To assess internalizing problems, we used the subscales of the Youth Self-Report [1, 29]. Three subscales (i.e., anxious/depressed, somatic complaints, and withdrawn) were adopted to yield a composite score of internalizing problems in the current study [30]. An example item is, “I feel too fearful or anxious.” A 4-point Likert scale from 1 (*definitely not true*) to 4 (*definitely true*) was applied in this study. We calculated the mean score across three subscales, with a higher score representing greater internalizing problems. Previous studies have reported excellent internal consistency of the Youth Self-Report among Chinese youth [5]. In this study, Cronbach’s alpha was 0.93 for both groups of adolescents.

## Teacher autonomy support

To assess teacher autonomy support, we used a 9-item Learning Climate Questionnaire [31]. One example item is, “The head teachers tried to understand how we see things before suggesting new ways to do things.” A 5-point Likert scale from 1 (*strongly disagree*) to 5 (*strongly agree*) was used. We calculated the mean score of these nine items, with a higher value representing higher teacher autonomy support. Previous studies have shown a good reliability coefficient of this questionnaire among Chinese youth [32, 33]. In the present study, Cronbach’s alpha was 0.92 for both groups of adolescents.

## Personality traits

To assess personality traits, we used the Big Five Inventory [34, 35]. This inventory consists of 44 items and five dimensions: openness, conscientiousness, extraversion, agreeableness, and neuroticism. An example item is, “Does a thorough job (conscientiousness).” A 5-point Likert scale from 1 (*strongly disagree*) to 5 (*strongly agree*) was used. The mean score of each personality dimension was calculated separately, with a higher score representing greater tendencies to exhibit the targeted personality traits. Prior research showed good internal consistency of this inventory in Chinese populations [34]. In this study, for adolescents who experienced parental divorce, Cronbach alphas were 0.81, 0.86, 0.85, 0.79, and 0.78 for openness, conscientiousness, extraversion, agreeableness, and neuroticism, respectively; for adolescents who remained in intact families, the corresponding alphas were 0.81, 0.86, 0.86, 0.80, and 0.81.

## Family structures

In the current study, teachers sent the URL for an online data collection site to parents, asking for their current family structures. If parents reported that they were divorced, a few complementary questions were followed up to understand adolescents’ ages when parents got divorced and their present residential status. These items have been widely used in previous studies concerning post-divorce adolescent adaptation [36, 37].

## Confounding variables

Adolescents were required to answer a few items indicating their sociodemographic characteristics, including age, gender, and family socioeconomic status (SES). We statistically controlled for these variables, as they potentially influence adolescents’ internalizing problems [38, 39]. In addition, adolescents answered a 16-item Responding Desirably on Attitudes and Options Scale [40], assessing their social desirability. This is because internalizing problems are assumed to be culturally sensitive with self-reports [41]. The Cronbach’s alpha for this scale was 0.86 for both groups of adolescents in this study.

## Data analyses

Data analyses were performed in SPSS 27.0 [42], Mplus 8.0 [43], and R [44]. Missing values in the data set were first assessed by descriptive statistics. Cases with more than 20% missing data in one of variables of interest were omitted from subsequent analyses. Second, preliminary analyses supported the assumption that missing patterns in the current study were completely at random [45]. Thus, we applied an expectation-maximization algorithm to replace these missing values.

Concerning RQ1, an analysis of covariance (ANCOVA) was used to compare internalizing problems in adolescents whose parents got divorced and in adolescents who remained in intact families, after adjusting for age, gender, family SES, residential status, and social desirability.

Concerning RQ2, a latent profile analysis was conducted to derive personality profiles based on the Big Five personality indicators. The best profile solution was selected based on model fit indices (e.g., low AIC, BIC, and aBIC; significant likelihood ratio tests; high entropy), interpretability, and theoretical considerations [43, 46, 47].

With regard to RQ3, a linear regression model was used to address the direct and interactive associations of teacher autonomy support, personality profiles, and family structures with adolescents’ internalizing problems. We conceptually treated teacher autonomy support as an independent variable, internalizing problems as a dependent variable, and personality profiles and family structures as moderators. To establish interaction terms in the linear regression, we multiplied teacher autonomy support by each of the moderators (two-way interaction), both moderators simultaneously (three-way interaction), and the multiplication between two moderators. While interpretation and establishment of the interaction terms between two moderators were not our research focus, we had to insert this into the model as a prerequisite to creating the three-way interaction terms. Furthermore, we used simple slope analyses and visualized figures to probe the nature of significant interactive patterns [48, 49].

## Results

### Preliminary analyses and correlation matrices

Before addressing our RQs, we computed descriptive statistics, including means, standard deviations, and bivariate correlations, separately for adolescents from divorced families (see Table 1) and intact families (see Table 2).

**Table 1:** Descriptive statistics and bivariate correlations of study variables for adolescents who experienced parental divorce

Variables	*M*	*SD*	Range	1	2	3	4	5	6	7	8	9	10	11	12	13
1. TAS	3.65	0.82	1–5	-												
2. Openness	3.64	0.75	1–5	0.21^**^	-											
3. Conscientiousness	3.48	0.74	1–5	0.31^***^	0.45^***^	-										
4. Extraversion	3.46	0.76	1–5	0.19^**^	0.48^***^	0.31^***^	-									
5. Agreeableness	3.99	0.60	2–5	0.24^***^	0.35^***^	0.56^***^	0.43^***^	-								
6. Neuroticism	2.87	0.87	1–5	− 0.11	0.05	− 0.05	− 0.20^**^	− 0.13^*^	-							
7. Internalizing problems	1.95	0.67	1–4	− 0.25^***^	− 0.15^*^	− 0.17^**^	− 0.46^***^	− 0.26^***^	0.68^***^	-						
8. Age	15.56	1.58	13–18	0.04	0.01	− 0.01	− 0.07	0.06	0.05	0.02	-					
9. Gender ^a^	-	-	1–2	− 0.01	− 0.07	0.04	− 0.06	0.08	− 0.01	− 0.01	0.08	-				
10. Socioeconomic status	− 0.03	3.64	-7.31-9.88	− 0.13	0.04	0.03	0.10	0.01	0.04	0.05	− 0.06	− 0.07	-			
11. Residential status ^b^	-	-	1–4	0.04	0.11	0.04	0.04	0.12	− 0.04	− 0.06	− 0.22^***^	− 0.08	− 0.08	-		
12. Social desirability	4.23	0.35	1–7	0.22^***^	0.07	0.13^*^	0.11	0.27^***^	− 0.12^*^	− 0.09	0.05	0.03	− 0.17^**^	0.09	-	
13. Post-divorce Years ^c^	7.91	4.05	1–17	0.05	0.04	0.01	0.14^*^	0.06	− 0.04	− 0.01	− 0.25^***^	0.02	− 0.03	0.01	0.05	-

*Note*. TAS = teacher autonomy support. ^a^ coded as 0 = boys, 1 = girls; ^b^ coded as 1 = residing with biological fathers, 2 = residing with biological mothers, 3 = residing with other relatives or grandparents, and 4 = parents share custody; and ^c^ Years refer to the years since parents got divorced

* *p* < .05 ** *p* < .01 *** *p* < .001

**Table 2:** Descriptive statistics and bivariate correlations of study variables for adolescents who remained in intact families

Variables	*M*	*SD*	Range	1	2	3	4	5	6	7	8	9	10	11	12
1. TAS	3.69	0.79	1–5	-											
2. Openness	3.71	0.70	1–5	0.16^***^	-										
3. Conscientiousness	3.65	0.68	1–5	0.29^***^	0.46^***^	-									
4. Extraversion	3.51	0.75	1–5	0.22^**^	0.54^***^	0.43^***^	-								
5. Agreeableness	4.01	0.59	2–5	0.27^***^	0.45^***^	0.59^***^	0.53^***^	-							
6. Neuroticism	2.68	0.86	1–5	− 0.12^***^	0.04^*^	− 0.12^***^	− 0.16^***^	− 0.15^***^	-						
7. Internalizing problems	1.78	0.62	1–4	− 0.20^***^	− 0.09^***^	− 0.29^***^	− 0.35^***^	− 0.26^***^	0.59^***^	-					
8. Age	15.66	1.57	13–18	0.02	0.01	− 0.01	− 0.01	0.05^**^	0.05^**^	0.02	-				
9. Gender ^a^	-	-	0–1	− 0.01	− 0.09^***^	0.03	− 0.02	0.06^**^	0.06^***^	0.05^**^	0.01	-			
10. Socioeconomic status	− 0.01	3.66	-8.16-13.46	− 0.05^**^	0.08^***^	0.02	0.07^***^	0.04^*^	− 0.01	− 0.02	− 0.02	− 0.01	-		
11. Residential status ^b^	-	-	1–4	0.07^***^	0.02	0.04^*^	0.02	0.03	− 0.03	− 0.05^**^	0.02	0.03	− 0.04^*^	-	
12. Social desirability	4.30	0.33	1–7	0.13^***^	0.06^**^	0.13^***^	0.11^***^	0.17^***^	− 0.10^***^	− 0.12^***^	− 0.07^***^	0.05^**^	− 0.05^**^	0.01	-

*Note*. TAS = teacher autonomy support. ^a^ coded as 0 = boys, 1 = girls; and ^b^ coded as 1 = residing with biological fathers, 2 = residing with biological mothers, 3 = residing with other relatives or grandparents, and 4 = parents share custody

* *p* < .05 ** *p* < .01 *** *p* < .001

As depicted in Tables 1 and 2, for both groups of adolescents, teacher autonomy support, openness, conscientiousness, extraversion, and agreeableness were each negatively and significantly associated with internalizing problems, whereas neuroticism was positively related to internalizing problems. In addition, Table 2 presents some significant associations of confounding variables with the outcome: girls reported higher internalizing problems than did boys; adolescents living with their fathers or mothers only reported higher levels of internalizing problems than did those living with both parents; and social desirability was negatively related to internalizing problems.

## RQ1: Group differences in internalizing problems

**Fig. 1:**
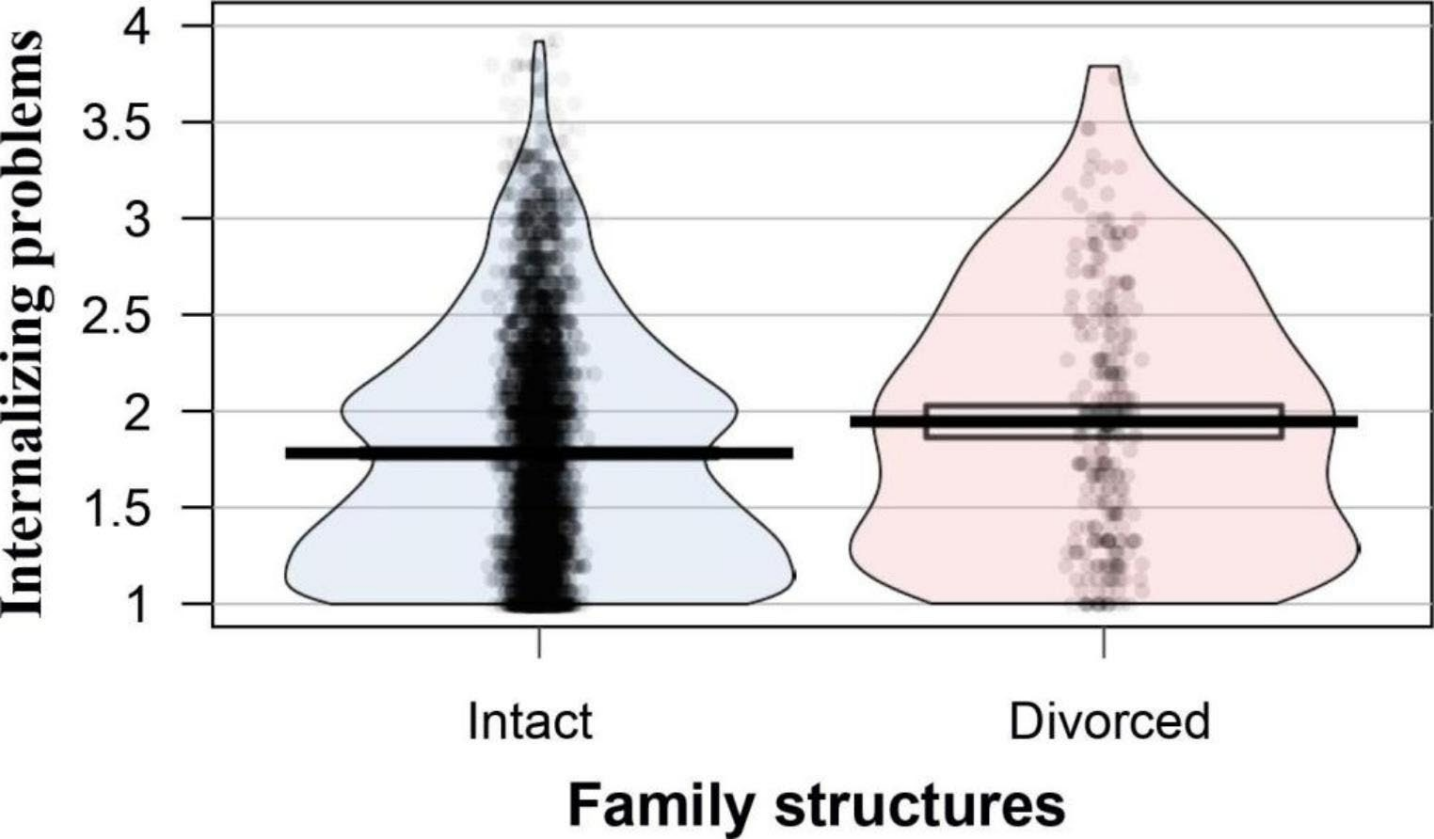
Group differences in internalizing problems *Note. N* = 2756. Points - Raw data, Bar / Line – Mean, Bean - Data distribution, Band - Confidence interval

To examine RQ1, we conducted an ANCOVA with internalizing problems as a dependent variable and family structures (divorced vs. intact families) as an independent variable. The results, after adjusting for covariates, indicated that adolescents who experienced parental divorce reported higher internalizing problems than did adolescents who remained in intact families, but the difference was marginally significant, *F* = 3.64, *p* = .05 (see Fig. 1).

## RQ2: identification of personality profiles

To examine RQ2, we conducted a latent profile analysis to identify possible personality profiles. Table 3 reports the model fit indices for different personality profiles.

**Table 3:** Model fit indices for different latent personality profiles

Profile	AIC	BIC	aBIC	Entropy	LMR-LRT	BLRT	Smallest profiles (%)
1-	30025.92	30085.14	30053.37	-	-	-	-
2-	27644.35	27739.10	27688.26	0.71	2344.25^***^	2393.57^***^	44.9%
**3-**	**26944.00**	**27074.28**	**27004.38**	**0.73**	**697.67** ^***^	**712.35** ^***^	**21.8%**
4-	26780.24	26946.05	26857.08	0.78	172.14	175.76	0.4%
5-	26583.51	26784.85	26676.82	0.75	204.43	208.73	5.2%

*Note*. *N* = 2756. The optimal model is highlighted in bold type. AIC = Akaike information criteria, BIC = Bayesian information, aBIC = Adjusted Bayesian information, LMR-LRT = Lo-Mendell-Rubin adjusted likelihood ratio test, BLRT = Bootstrapped likelihood ratio test

^**^p < .01, ^***^p < .001

As presented in Table 3, the likelihood ratio tests (i.e., the values of LMR-LRT and BLRT) were significant for the solutions with two or three profiles; however, these tests were insignificant for the four- and five-profile solutions, indicating that additional profiles after the three-profile solution did not offer significant explanatory power. In comparison with the two-profile option, the three-profile option exhibited lower levels of AIC, BIC, and aBIC; higher entropy; and theoretical coherence [24, 46]. We, therefore, regarded the three-profile solution as optimal for subsequent analyses.

**Fig. 2:**
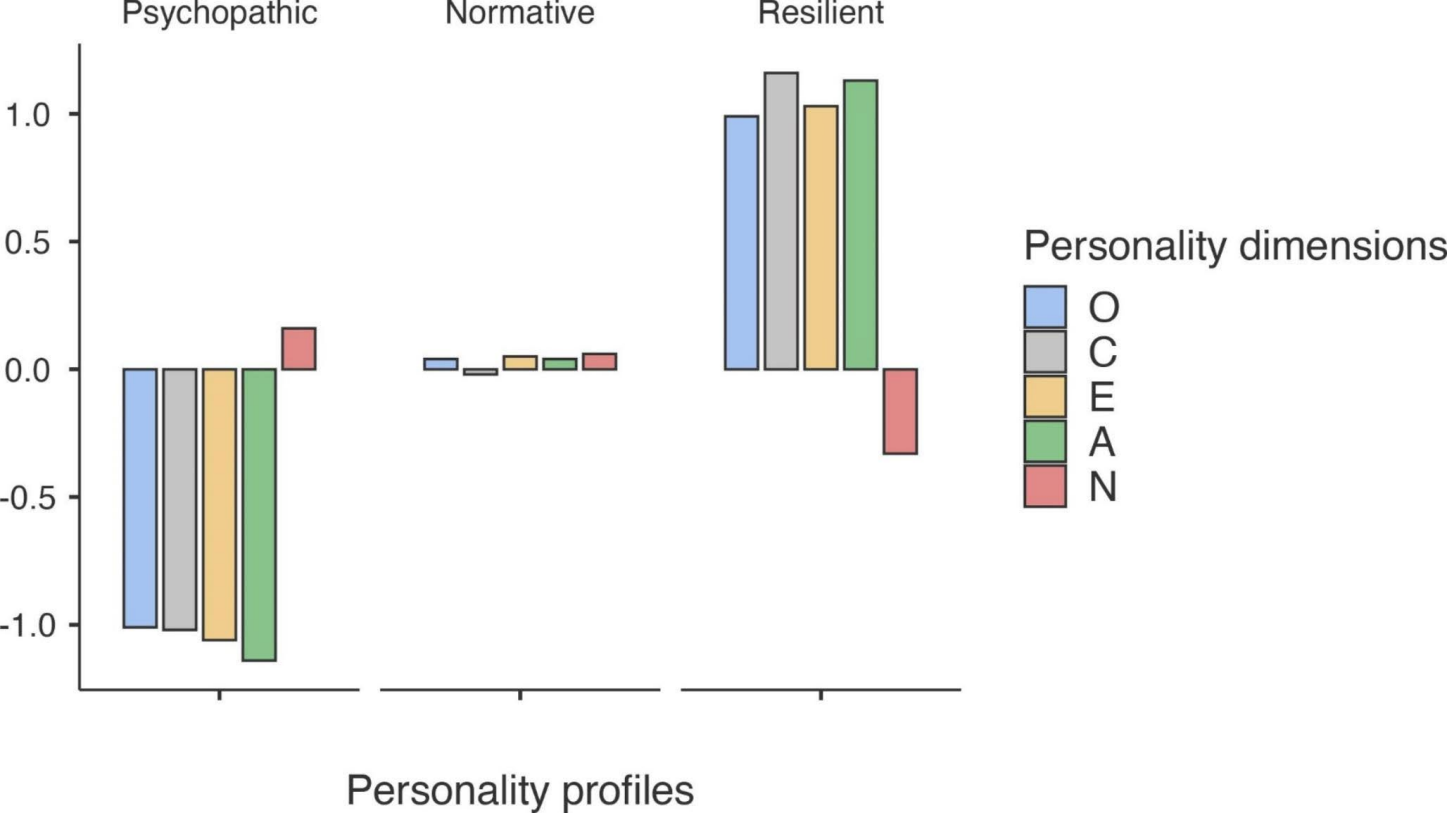
Three personality profiles based on the Big Five model of personality (z-standardized means) *Note. N* = 2756. Openness = O, Conscientiousness = C, E = Extraversion, A = Agreeableness, and N = Neuroticism

Specifically, these three profiles were characterized by the following features: adolescents in the first group were characterized by relatively high scores in neuroticism and low values in the remaining four personality dimensions, and thus this group was labeled as “psychopathic profile” (*n* = 625; 22.7%). Adolescents in the second group exhibited average scores in all five personality dimensions, and therefore this group was named “normative profile” (*n* = 1555; 56.4%). Adolescents in the last group showed low scores in neuroticism and high values in the remaining four personality dimensions, and thereby the label of “resilient profile” (*n* = 576; 20.9%) was assigned to this group. A visualized figure of these three personality profiles can be seen in Fig. 2.

## RQ3: Associations of teacher autonomy support, personality profiles, and family structures with internalizing problems

To examine RQ3, we performed a linear regression analysis, and the results are reported in Table 4. The model overall explained 13.1% of the variance in internalizing problems (residual *df* = 2735, AIC = 4893.69, and *χ*^2^/*df* = 0.34).

**Table 4:** Regression analysis predicting internalizing problems from teacher autonomy support, personality profiles, family structures, covariates, as well as interaction effects

Variables	*b*	*b* SE	95% CI for *b*		*t*	*p*
Teacher autonomy support (TAS)	-0.12	0.03	-0.18	-0.05	-3.65	< 0.001
Psychopathic vs. normative	-0.24	0.05	-0.33	-0.14	-4.74	< 0.001
Psychopathic vs. resilient	-0.56	0.07	-0.70	-0.43	-7.96	< 0.001
Normative vs. resilient	-0.33	0.06	-0.45	-0.20	-5.23	< 0.001
Family structures ^a^	0.09	0.05	-0.01	0.20	1.76	0.08
Age	0.01	0.01	0.00	0.02	1.41	0.16
Gender ^b^	0.07	0.02	0.03	0.11	3.08	0.01
Socioeconomic status	0.00	0.00	-0.01	0.00	-0.88	0.38
Residential status 1^c^	0.11	0.05	0.02	0.21	2.41	0.02
Residential status 2	0.04	0.05	-0.05	0.13	0.91	0.36
Residential status 3	-0.04	0.06	-0.17	0.08	-0.67	0.50
Social desirability	-0.12	0.03	-0.19	-0.06	-3.58	< 0.001
TAS x psychopathic vs. normative	-0.09	0.06	-0.21	0.03	-1.52	0.13
TAS x psychopathic vs. resilient	-0.13	0.09	-0.31	0.04	-1.49	0.14
TAS x normative vs. resilient	-0.04	0.08	-0.20	0.12	0.00	0.62
TAS x family structures	-0.04	0.06	-0.16	0.09	-1.00	0.56
Family structures x psychopathic vs. normative	-0.07	0.10	-0.26	0.13	-0.67	0.50
Family structures x psychopathic vs. resilient	-0.14	0.14	-0.42	0.13	-1.00	0.31
Family structures x normative vs. resilient	-0.07	0.12	-0.32	0.17	-1.00	0.55
TAS x psychopathic vs. normative x family structures	-0.28	0.12	-0.52	-0.04	-2.33	0.02
TAS x psychopathic vs. resilient x family structures	-0.26	0.18	-0.60	0.09	-1.45	0.15
TAS x normative vs. resilient x family structures	0.02	0.16	-0.29	0.34	0.14	0.89

*Note*. *N* = 2756. ^a^ coded as 1 = adolescents who experienced parental divorce, 0 = adolescents who remained in intact families; ^b^ coded as 0 = boys, 1 = girls; and ^c^ coded as 1 = residing with biological fathers, 2 = residing with biological mothers, 3 = residing with other relatives or grandparents, and 4 = parents share custody. R^2^ = 13.3%

As shown in Table 4, teacher autonomy support was negatively associated with internalizing problems. Moreover, the three-way interaction term (teacher autonomy support x personality profiles [psychopathic vs. normative] x family structures) was negatively associated with internalizing problems.

**Fig. 3:**
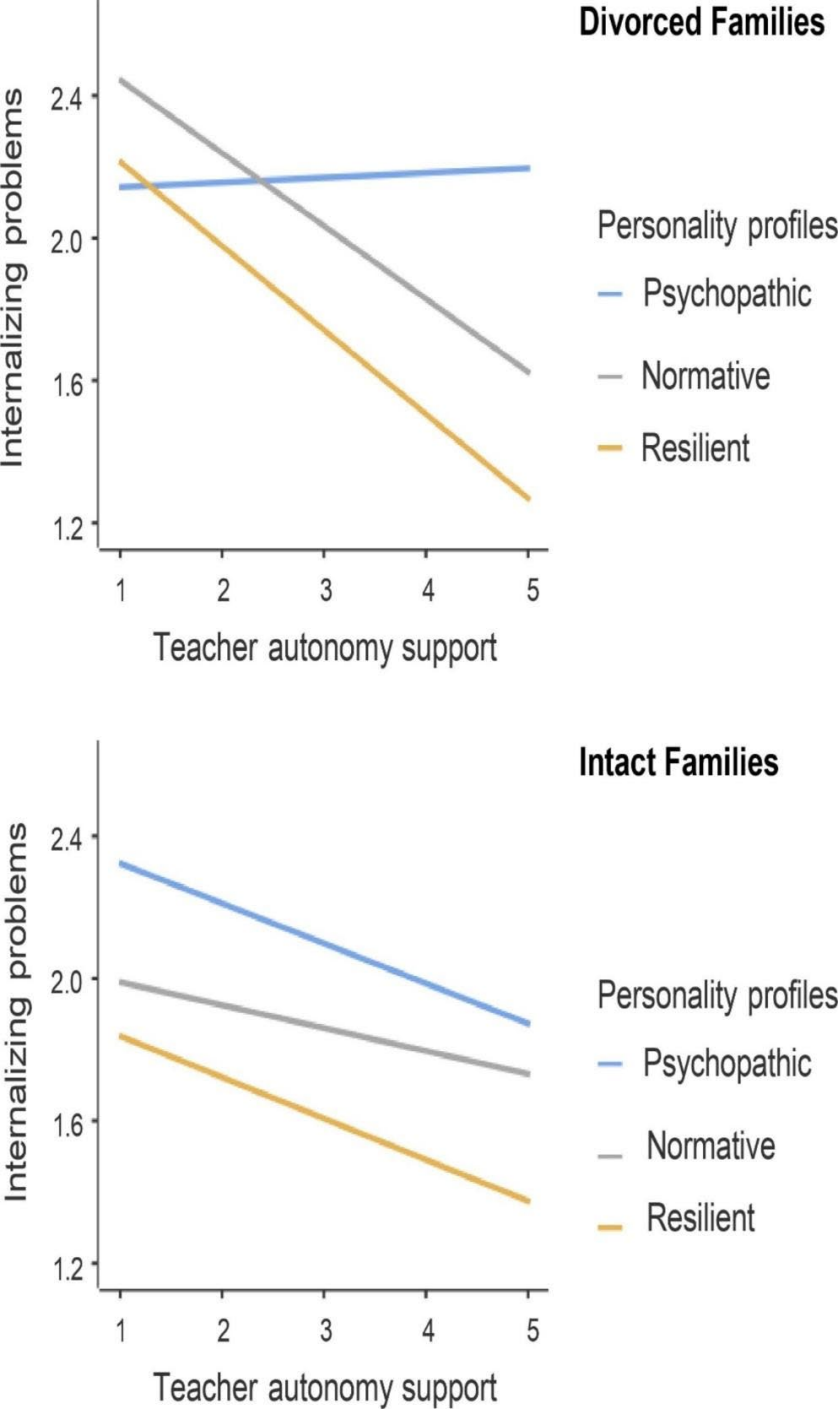
Interaction effect of teacher autonomy support, personality profiles, and family structures on internalizing problems *Note. N* = 2756

Furthermore, simple slope analyses (see Fig. 3) revealed that for adolescents from divorced families, the association among teacher autonomy support and internalizing problems was significantly negative for the normative profile (*b* = -0.20, *SE* = 0.07, 95%CI for *b* [-0.33, -0.07], *t* = -3.11, *p* < .001) but not for the psychopathic profile (*b* = 0.03, *SE* = 0.09, 95%CI for *b* [-0.15, 0.21], *t* = 0.32, *p* = .75). By contrast, for adolescents from intact families, the negative association between teacher autonomy support and internalizing problems remained significant in both normative (*b* = -0.11, *SE* = 0.03, 95%CI for *b* [-0.18, -0.05], *t* = -3.28, *p* < .001) and psychopathic profiles (*b* = -0.06, *SE* = 0.02, 95%CI for *b* [-0.11, -0.02], *t* = -3.11, *p* < .001).

## Discussion

Given the increasing divorce rates globally, the current study compared internalizing problems of adolescents from divorced and intact families and investigated the association of teacher autonomy support with adolescents’ internalizing problems, as well as ascertained individual differences of this association by postulating moderation by personality profiles using a person-centered approach and family structures. In accordance with research hypotheses, the current findings showed that adolescents who experienced parental divorce reported higher internalizing problems than did their peers who remained in intact families. Moreover, three personality profiles were found: psychopathic, normative, and resilient. In addition, teacher autonomy support was negatively associated with adolescents’ internalizing problems for the whole sample, but interaction analyses further demonstrated that this negative association was significant for psychopathic and normative adolescents from intact families; in contrast, this negative association was significant only for normative adolescents from divorced families, as compared with their psychopathic peers.

With regard to RQ1, we compared adolescents’ internalizing problems from different family structures. Consistent with the first hypothesis, the results confirmed that adolescents whose parents got divorced reported higher internalizing problems than did those who remained in intact families. One possible interpretation is related to sociocultural norms in China, emphasizing interdependent relationships and traditional two-parent families [5, 41] Indeed, marital dissolution is often regarded as precipitating the loss of the whole family’s dignity. Such family transitions often cause economic deprivation and diminished parental involvement, which subsequently exacerbate the impact of parental divorce on adolescents’ well-being [51]. Another potential interpretation could be that adolescents’ cognitive maturity may increase their ability to understand the underlying reasons for parental divorce. Adolescents’ cognitive maturity may also lead parents to rely on their offspring to provide emotional support and advice, which puts excessive pressures and responsibilities on adolescents [52]. Likewise, adolescents generally experience rapid and intense biological and socioemotional changes, so diminished parental involvement after parental divorce may decrease adolescents’ immediate support networks. Such decreases then result in heightened levels of psychological distress among adolescents already facing other salient changes and transformations [53]. However, to be noted, although adolescents whose parents got divorced did report higher internalizing problems than those who remained in intact families, the results were marginally significant. As indicated by prior research [54], one possible explanation could be related to the features of the current sample. In this study, parental divorce on average was not recent, and thus this adverse effect on adolescents’ emotional dysfunctions might be somehow reduced.

With regard to RQ2, this study revealed three personality profiles: psychopathic, normative, and resilient. These results replicated only the resilient profile based on Block and Block’s (1980) framework, but unexpectedly, the remaining profiles did not resemble the overcontrolling or undercontrolling profiles. This corroborates prior research [23, 28, 55] indicating that overcontrollers and undercontrollers are not unambiguously interpretable. One possible explanation could be related to the questionnaire of the Big Five personality dimensions we employed in this study, a measurement which is distinct from those used in previous studies [26, 56]. As demonstrated by prior research [57], the replicability of these three profiles is highly sensitive to measures and informants. The average age of the adolescent samples under investigation may also help explain the inconsistent finding from prior research. For instance, Soto and colleagues (2011) have demonstrated that adolescents around 15 years old exhibit the lowest levels of agreeableness and conscientiousness among all age groups, but the highest levels of neuroticism [58]. This finding indicates that youth during this life period are confronting “storm and stress” due to significant biological and socioemotional changes and subsequent related challenges. In accordance with these developmental features, in this study, the psychopathic profile is characterized by a mixture of low agreeableness, low conscientiousness, and high neuroticism. This indicates that adolescents in this profile may exhibit high emotional instability, social deviance, and impulsivity. Considering these features, we named it the psychopathic profile, as suggested by prior research [59]. Moreover, the normative profile characterized by average scores across all personality dimensions represents the largest percentage of the whole sample. One possible interpretation could be related to Zhong-Yong thinking (the Doctrine of the Mean) in Chinese societies. Under this critical philosophical thought, individuals tend to avoid extremities and tolerate contradictions [60]. Such tendencies also align with adolescents’ developmental features. Socio-cognitive maturity during adolescence, for instance, may lead youth to think from multiple perspectives to balance apparent conflicts and integrate the self and external situations to avoid extremes in self-reporting related to specific personality characteristics [53]. Therefore, most adolescents reported an average level of scores across all personality dimensions, and a similar pattern was reported in previous studies on Chinese adolescents [61]. Although interesting, this finding is circumscribed by an methodological artifact because all the measurements in the current study were conducted by self-reported formats, and thus adolescents tended to report on all items based on average levels. This finding, however, requires further confirmation and replication.

With regard to RQ3, we investigated the association of teacher autonomy support with adolescents’ internalizing problems and the moderating role of these emerging personality profiles and family structures in this association. Following the third hypothesis, the current study supported a negative relationship between teacher autonomy support and adolescents’ internalizing problems. Despite some debates, this study further enriches prior research [14, 33, 62, 63], suggesting the benefits of autonomy support on adolescents’ optimal functions in a cultural context that is more collectivistic and authoritarian in its traditional orientation. One possible explanation, based on self determination theory, is that adolescents naturally tend to self-organize initiatives and self-endorsed perspectives and that autonomy is regarded as one of the core aspects of optimal emotional functions [18]. Autonomy-supportive ways of interacting with adolescents (e.g., asking students what they want, providing rationales for requests, and listening carefully to students’ agendas) can facilitate their intrinsic motivation, which is linked to less internalizing difficulties. Moreover, during adolescence, youth strive to seek greater autonomy from adult authority figures, such as teachers, due to the development of the self and increased individuation and independence [53]. The high level of autonomy support provided by teachers during that developmental context may fulfill adolescents’ increased autonomous motivation and alleviate their emotional distress, leading adolescents to therefore report low internalizing problems [33].

In addition, interaction analyses revealed interesting individual differences in this association. The negative association under investigation remained significant for both psychopathic and normative adolescents from intact families. In contrast, this negative association was significant only for normative adolescents from divorced families. One possible interpretation could be that adolescents from intact families may establish a secure base relationship with their parents and learn a set of social skills and the reciprocal nature of social interactions, which they can then apply to their interactions with teachers [64, 65]. In this regard, autonomy-supportive teachers would become significant figures counteracting potential difficulties and psychological distress for adolescents, regardless of their personality traits. Nevertheless, for adolescents from divorced families, physical communication with their parents, particularly with non-residential parents, becomes challenging and may decrease over time. Yet parents undeniably still play an essential role in adolescents’ emotional functions despite the increasing independence and autonomy youth experience during adolescence [53]. Likewise, such a family transition may cause the loss of original networks with teachers and peers, which brings an additional challenge to adolescents’ emotional energy when handling the challenges caused by parental divorce [66]. These dynamics may be particularly problematic in adolescence, a time when youth are confronting salient biological and socioemotional changes [53]. For adolescents, extrafamilial contexts (e.g., teachers) are vital for conveying emotional comfort and providing positive guidance for adolescents’ psychosocial competence in the face of parental divorce. Not surprisingly, psychopathic adolescents who are emotionally constrained and behaviorally inhibited show low adaption flexibility and, therefore, may have some hesitations when interacting with their teachers [25]. In this perspective, although teacher autonomy support is intended to be attentive and helpful, psychopathic adolescents may still be unwilling to share their emotional needs or deal with their psychological distress appropriately.

Despite these valuable findings, a few notable limitations should be considered. First, the current study was based on a cross-sectional design, and thus the temporal order of study associations cannot be inferred. Future studies should employ a prospective longitudinal design to elucidate this temporal order of studied associations. Second, the current study was solely based on a quantitative approach leveraging only self-report questionnaires, although the sample size was large and the measurements employed were previously validated in Chinese adolescents. This methodological limitation should be avoided in future studies by adopting a multi-method approach (e.g., obtaining parents’ ratings on adolescents’ emotional functions and examining how teachers evaluate their autonomy support for students) and/or a mixed-method approach. Importantly, a mixed-method approach can capitalize on the advantages of both quantitative and qualitative approaches while offsetting the drawbacks of each. For instance, by employing diary accounts or in-depth interviews, future researchers could deeply probe and gain valuable insights into the complex interplay that contributes immensely to our understanding of how adolescents respond to parental divorce. Finally, the present study was based on a monocultural dataset, and thus the generalizability of research findings to other countries/cultural contexts may be restricted. A cross-cultural investigation of the studied association is warranted in forthcoming studies.

## Summary

Building on a large sample size of adolescents, the present research reconfirms the contrasting evidence in the literature, suggesting that adolescents from divorced families are vulnerable, particularly in terms of internalizing problems, and that three personality typologies (i.e., psychopathic, normative, and resilient) are probably the optimal profile solutions. Furthermore, the finding that autonomy support mitigated adolescents’ internalizing behavior confirms the universal benefits of such support in a collectivistic culture. This study points to the importance of facilitating an autonomy-supportive, student-centered teaching style in helping adolescents deal with emotional distress. Nevertheless, individual analyses of this association further indicate that mental health professionals and school counselors should pay psychopathic adolescents who experienced parental divorce exceptional attention.
